# The Soul and the Body in the Philosophy of the Rambam

**DOI:** 10.5041/RMMJ.10040

**Published:** 2011-04-30

**Authors:** Avshalom Mizrahi

**Affiliations:** Academic Director and Head of the Faculty of Naturopathy, Reidman International College for Complementary Medicine, Tel Aviv, Israel

**Keywords:** Maimonides, Rambam, soul, body, soul-body

## Abstract

Among the wide-spectrum contribution of the Rambam – the Maimonides – in philosophy to the word and to Judaism are his ideas on the body and on the soul and on the relations between them. His major approaches in these subjects are the following: 1) The body is the home of the soul, and the soul guides the body. That means the body and the soul are one unit. 2) The soul has five virtual parts. Each part is responsible for another activity in the human being. 3) Except for the treatment of diseases of the body and the soul with drugs, foods, physical exercise, etc., the Rambam believes that maintaining the health – of the body and of the soul – lies first of all, and probably exclusively, in observing the commandments and improving one’s ways, morals and conduct up to their highest levels, toward all of the world’s creatures. 4) The Rambam is of the opinion that one needs to persist in learning the Torah. One should worship God with awe and love and observe good values and virtues. All of these build the frameworks that maintain mental health and strengthen man’s abilities to develop skills for maintaining bodily health. This is so because body and soul are one – which is the basis of the Rambam’s philosophy of health and medicine.

**“Moses son of Maimon knew to bring health to both body and soul.”**

*(Al Sa’id Ibn Sina Maluch, Arab physician and poet, a contemporary of the Rambam)*

The Rambam’s ideas on body and soul constitute a very important foundation in the philosophy and the understanding of health and medicine. They have become not only part of Jewish heritage, but have exerted influence and have been applied on a universal level.

## ABOUT THE RAMBAM

Rabbi Moshe Ben Maimon (Moses Maimonides), known by the acronym “the Rambam”, was born on 14 Nissan 4898 (28 March 1138) in Cordova, Spain. He died on 20 Tevet 4965 (13 December 1204) in Fostat, as Cairo was called in ancient times.

During his life and to date, the Rambam is considered as one of the greatest and most important philosophers and intellectuals ever. He was a scientist, physician, scholar, spiritual leader, and was among the greatest rabbinical arbiters, if not the greatest of them all.

His sublime personality, his clear thinking in his interpretation and comprehension of the Torah in all its smaller and larger aspects, his ability to deeply understand and explain to the reader all the Jewish laws as well as the mysteries and powers of the human mind, his understanding of the human body and human morals – both the Jewish and universal ones – are extremely impressive. These and other skills made the Rambam into one of the most revered persons ever – mainly among Jews but also among people of other beliefs and nations.

Thanks to his talents, the Rambam earned the name of the great eagle ( 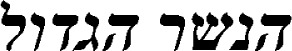). Thanks to his sublime personality, the well known saying “From Moses (who received the Torah) till Moses (the Rambam) there was no one like Moses” was coined.

The Rambam was known as a pre-eminent philosopher, both in Arab culture and European culture. In Arabic, the Rambam is known by his full name, Abu Imran Musa Bin Maimun Bin Abed Allah Al-Kurdubi Al-Israili – Moses son of Maimon, Jewish inhabitant of Cordova. His shorter name in Arabic is “Musa Ibn Maimon”. In the European languages he was called Maimonides, which in Greek means Son of Maimon.

## THE BOOKS OF THE RAMBAM ON HEALTH AND MEDICINE – A TREASURE OF UNIVERSAL IMPORTANCE

The rationalistic teachings of the Rambam are expressed in the abundance of books he wrote. His understanding and philosophy of the Jewish laws, his vision of the mosaic of life and morals, his knowledge of the structure of body and soul and the relation between them, the principles of health and the knowledge of curing diseases – all of these are reflected in his many works. The Rambam wrote not only philosophical and religious teachings, but also books on the structure of the soul and the human body, and on matters of health, medicine, and diseases.

The following are the most important books written by the Rambam, dealing with philosophical issues on the behavior, the constitution, and the health of man. The order of their appearance in this list is of no importance, all of them are equally important.

### Moreh Nevuchim ( 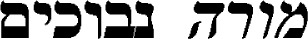) – Guide for the Perplexed

This is the most elaborate philosophical-religious book written by the Rambam. It has become an inalienable part of Jewish heritage and philosophy.

### Mishneh Torah ( 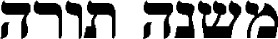) – Second to the Torah

This is the crowning of the Rambam’s in-depth understanding of the Torah laws, and therefore this work was called Mishneh Torah. Others preferred to call this book Sefer Hayad Hachazaka ( 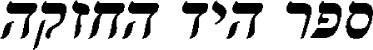), Book of the Strong Hand. The origin of this name may lie in the fact that this elaborated work comprises 14 volumes (14 reflected in Hebrew letters is 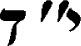 which can also be read as “yad”, which in Hebrew means hand). In the volume Mada ( 
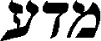
 – The Book of Knowledge), chapter Hilchot De’ot ( 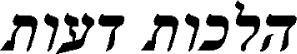 – Laws of Behavior), the Rambam expounds his vision on personal development and the health of body and soul.

### Shemona Perakim ( 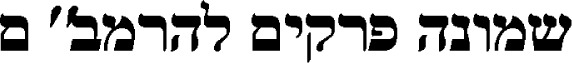) – The Eight Chapters

This book is the Rambam’s introduction to the Tractate of the Fathers ( 
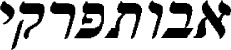
). In this work, the Rambam focuses mainly on the recesses of the human soul and body.

### Regimen of Health ( 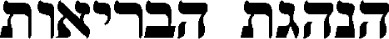)

This is a comprehensive work on matters of health and healing. Here, the Rambam elaborates on the psychosomatic relation between a person’s mental state and his physical sensations. In our era, this phenomenon is known as the “mind–body connection”.

### Chapters of Moses in Medicine ( 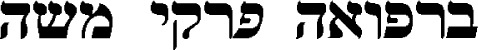)

This book contains a compilation of some 1,500 health directives on all fields of medicine. The contents of the book are mostly founded on the teachings of Galen, the well known physician and philosopher of Greek descent who lived in Rome around the year 200.

### Commentary on the Aphorisms of Hippocrates ( 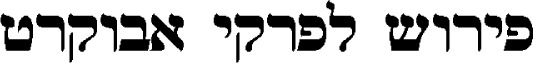)

The Rambam was a great admirer of the teachings on medicine and diseases of Hippocrates, who was the greatest physician of ancient Greece. The book describes essential principles of Hippocrates on the approach to life, health, medicine, and diseases and how the Rambam sees them. The book focuses on matters of health, how to improve health, hygiene, etc.

### Extracts from Galen ( 
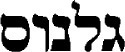
 ( 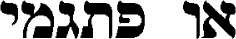) 
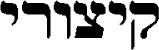
)

This book contains a well arranged compilation of Galen’s teachings. The Rambam compiled everything written by Galen about diseases and their healing, but this compilation includes only the treatment of diseases that were acceptable to the Rambam.

### Treatise on Hemorrhoids ( 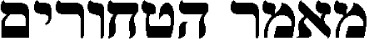)

The book is about diseases of the digestive tract and how to treat and avoid them.

### Treatise on Asthma ( 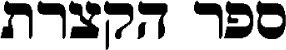)

In this book, the Rambam describes the disease and how to treat and avoid it.

### Glossary of Drug Names ( 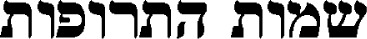)

This is a compilation of some 2,500 drugs and medical indications.

This is a very impressive list indeed, rare in its scope and depth, dealing with health, healing, and the understanding of the recesses of the soul and its connection with the body.

## THE SOUL IS THE MAIN COMPONENT OF THE HUMAN BEING

According to the Rambam’s philosophy, the soul is the main component of the human being. His writings present an in-depth research of the essence of the human soul, its structure, and its many functions. It is a philosophical analysis, the “branches” of which penetrate into the essence of life through the behavior of man and the framework for maintaining his health: the health of the soul is projected to the body.

This vision of the Rambam is anchored in what already has been written in the book Deuteronomy (4:15): “Take ye therefore good heed unto your souls.” The importance of “heeding the soul” – and not the body – is emphasized not only in the Jewish vision but also among other religions and nations. The psyche is the essence and controls all human physical, psychological, and spiritual functions.

## THE HUMAN SOUL IS ONE

“Know that the human soul is one” (chapter 1). This is the opening sentence of his book *The Eight Chapters.* This single soul regulates all the various actions of human existence. On this, the Rambam says: “It [the soul] has many diversified activities. Some of these activities have been called souls, which has given rise to the opinion that man has many souls.” (*The Eight Chapters*, chapter 1). In the first chapter of this book, the Rambam continues to elaborate on the issue of oneness of the soul.

According to the Rambam, every individual possesses various faculties, but this does not mean that a person has several identities. This view the Rambam had in common with other philosophers, such as the Greek philosopher Aristotle, Rabbi Sa’adia Ga’on (born in Egypt in 882/892, died in Baghdad 942, a prominent rabbi, philosopher, and exegete of the Geonic period), and Rabbi Shimon Ben-Tzemach Duran (1361–1444, physician, astronomer, mathematician, philosopher, and rabbinical authority).

The reader discovers an interesting facet in this comprehensive, holistic view of the Rambam. It means that the soul has various faculties, but its parts are not divided. The soul is one and contains the entire human essence.

The Rambam’s philosophical purpose, as he understands it, is to prevent people from entering into a situation of split personality. There is no external factor of any kind that reigns over all the faculties and abilities of man. Each person has one identity residing in one soul. Therefore, man cannot claim an eruption of uncontrollable powers inside of him, exempting himself from the responsibility of such eruptions. That is because they are his own powers and not a foreign influence originating from outside his body.

The “evil inclination” ( 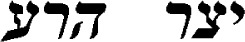) does not represent an external power taking control over a person, but this inclination is also part of a person’s personality. It has already been said in the book Genesis (8:21): “for the intent of man’s heart is evil from his youth”.

When the Rambam uses the term “soul”, he does not mean a mystical entity of any kind that controls a person from the outside, but he refers to the natural life-force residing in man, as the Rambam defines it in volume 1 of *Mishneh Torah*, Hilchot Yesodoth Hatorah ( 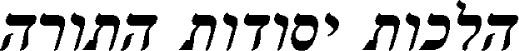) – the Fundamentals of the Torah (4:8): “... the soul resides in all living creatures, by which it eats, drinks, gives birth and feels and contemplates...”

The soul according to the Rambam is depicted as a natural life-force which encompasses the conscious ability of man to say “I”. This natural life-force generates man’s consciousness to a higher level of development, which gives man the ability to say “I”. By using these powers, man is able to understand that the saying “the soul is one” points to a reality of a higher quality that does not only refer to a convergence of the powers operating in man but to the unity of a person’s soul.

As said, the Rambam’s philosophical view refers to the existence of one soul, which is a complete unit containing everything occurring in the human psyche. True, the personality of a person is one, but in this personality one can discern several activities, all of which originate from the same soul. This view contradicts the view of other sages who existed among Jews and other nations over the generations. The latter saw man as “souls” and not as one soul.

Here are two examples of this view:

### The vision of Chasidic Chabad movement of the structure of the soul

Interesting is the following view, which at first sight seems to contradict the Rambam’s view that the individual human soul forms one unity. The view of the Chasidic Chabad movement (Chabad ( 
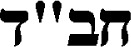
), acronym of the words “wisdom, understanding, knowledge”, is a wide-spread Chasidic movement) which is expressed in the book *Tanya* (the book of Tanya, written by Rabbi Schneur Zalman of Liadi, is the fundamental, classic work of the Chabad Chasidic philosophy), is that a person has two souls: a “beastly soul” and a “godly soul”. The latter expresses the belief that man should wish and strive for love and awe for the Almighty God.

In-depth examination of this view shows that what the Rambam considers to be the natural faculties of man inside the human soul – as expounded in *The Eight Chapters* – Chabad considers as the “beastly soul”. This means that the “godly soul” – which exists according to the Chabad teachings – does not contain what the Rambam calls the natural components of a person’s personality. This clarification shows that actually there is no contradiction, and, according the majority of sages in Israel, the human soul is one.

In the opinion of many physicians, man is made of three levels: the level of growth, the beastly level, and the intellectual level. They run parallel to various phenomena found in nature. On this basis, some people came to the conclusion that a man has three “souls”, but in the first chapter of *The Eight Chapters* the Rambam clarifies, time and again, that the human soul is one.

### Galen’s vision of the human soul

Galen – the well known physician – tells in the beginning of his book that his approach is based on Plato’s teachings. These teachings say that man has three souls: a natural, an essential, and an emotional soul. This is not the place to elaborate on verifying this view on the human soul, but it is appropriate to present it against the Rambam’s view of the soul as being one.

## SINCE THE SOUL CREATES THE BODY

The Rambam embraces all the material needs of man under the cover of sanctity of the soul. For him, this is a holy objective.

In the Rambam’s view, all the bodily activities, although looking like physical functions, are directed by the soul, which rules over the body and its various needs. Based on this vision, there is neither a need nor a power exerted from outside the body that is not managed and controlled by the human soul.

It is true that man is made up of a physical component and a mental component, but man unifies them into one entity. This is the basis for the Rambam’s words in the beginning of *The Eight Chapters:* “the human soul is one”. One and the same personality eats and drinks, is sad or happy, is doing gymnastics and sleeps, prays and fights. All those activities are generated by the same soul.

In this spirit, the very fact of obeying the commandments is medicine of the soul and not medicine (healing) of the body, yet the connection between them is so strong, that medicine of the soul, such as obeying the commandments, exerts influence on man’s physical health.

## THE SOULS OF ANIMALS DIFFER FROM THOSE OF HUMAN BEINGS

Although the soul of each and every animal is a whole unit as well, the powers at work in the soul of each of the world’s creatures are different and adapted to the nature of the animal. This view of his the Rambam described as follows in the first chapter of his book *The Eight Chapters*:

For the nutritive faculty by which man is nourished is not the same, for instance, as that of the ass or the horse – man is sustained by the nutritive faculty of the human soul, the ass thrives by the nutritive faculty of its soul, and the palm tree flourishes by the nutritive faculty peculiar to its soul. Although we apply the same term nutrition to all of them indiscriminately, nevertheless its signification is by no means the same.

The analysis of these differences in mental activity can be explained by the simple process of eating, which is seemingly a physiological process man has in common with all the world’s creatures. But that is not so. A predator eats meat because this is the food it needs, based on the natural demands of his body and soul. But if man eats meat, it offends his moral integrity although it is the same act of eating. However, the consequences for the human soul are different.

## THE FIVE FACULTIES OF THE SOUL

Although the soul is one, the Rambam divides it into five virtual parts or faculties. By doing so he follows in the footsteps of Aristotle.

The following are the five aspects of the soul:

**Ha-Zan ( 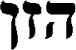) – the first, *nutritive,* part of the human soul.** This part runs parallel with the physical soul, or the “growing soul”, in the manner of the division of the soul by physicians. This part controls the physiological systems of the human body.
**Ha-Margish ( 
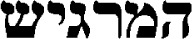
) – the second, *perceptive,* part.** This is not the part which in our era is referred to as “emotions”, but refers to the five wellknown senses: seeing, hearing, tasting, smelling, and touching.
**Ha-Medammeh ( 
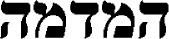
) – the third, *imaginative,* part.** In this part, the Rambam also included memory, which is at the basis of imagination. About this part, the Rambam says in the first chapter of *The Eight Chapters:*
  The imagination is that faculty which retains impressions of things perceptible to the mind after they have ceased to affect directly the senses which conceived them. This faculty, combining some of these impressions and separating others from one another, thus constructs out of originally perceived ideas, some of which it has never received any impression of, and which it could not possibly have perceived...
**Ha-Mit’orer ( 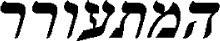) – the fourth, *appetitive,* part.** This part relates to the realm of feeling and sensations. It starts operating when dealing with emotional matters. This is what the Rambam says about it in *The Eight Chapters*, the first chapter:
  The appetitive is that faculty by which a man desires or loathes a thing, and from which there arise the following activities: the pursuit of an object or flight from it, inclination and avoidance, anger and affection, fear and courage, cruelty and compassion, love and hate, and other similar psychical qualities.
  This emotional part is connected with both the soul and the body. That is because this faculty of the soul makes use of the human body. This is an example of the mind–body connection in the human being.
**Ha-Maskil ( 
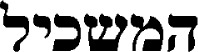
) – this is the fifth, *rational,* part,** the ability to engage in metaphysical ideas. The Rambam sees it as the part where things come together. In the first chapter of *The Eight Chapters*, he says that by means of this faculty “man knows things as they really are and which, by their nature, are not subject to change”. This view is based on the philosophy of Aristotle that everything existing in heaven and beyond is eternal. Metaphysics (literally: beyond nature) is the title of the book of Aristotle, which he wrote after his book Physics (nature). The book Metaphysics deals with matters beyond nature. By this is meant the things existing in the celestial orbs, the stars, the souls, and deity.

In the Rambam’s view, these five faculties form together the mosaic of the human soul with all its different functions.

There is a striking parallelism between the five parts of the soul according to the Rambam as mentioned above and the model of the five elements in traditional Chinese medicine – a model that forms one of the basic foundation theories.

This interesting parallelism is one of the elements of Yochi Keshet’s book *The Metamorphic Therapy* ( 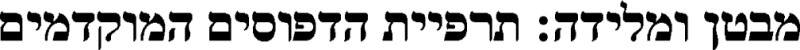).

## THE ESSENCE OF THE SOUL

Many wonder if there is such a thing, the essence of the soul. Nobody has ever seen nor touched it, and yet, almost everybody will confirm that all the world’s creatures have a soul.

The common belief is that the soul resides in the recesses of the brain, flows through the blood, and so reaches the various parts of the body.

Although we are unable to see or touch the soul, we see the effect of its actions. The soul shows itself in everything we say, in our smile, hug, look, and almost all our actions, not all of which are visible to others.

Most philosophers – among whom the Rambam – believe that the soul and the body complement one another, and some believe that they even protect one another.

However, it is clear to everybody that the different mental states affect our physical health. This is the extraordinary bond called the “mind–body connection”. This is the basic, classical view in the holistic understanding of life, health, medicine, and disease.

All expressions of the soul, happiness, sadness, pain, depression, joy, and many more occurring in the human soul, direct the manner in which the body will behave for better or worse – toward health or sickness.

## INTELLIGENCE IS A FORM OF THE SOUL

According to the Rambam’s view, the soul is a “matter”, which, in Hebrew, is related to the philosophical term “container”. Matter is what enables the appearance of “form”, or “reason”. On this the Rambam says in the first chapter of *The Eight Chapters*:

Know, however, that the soul, whose faculties and parts we have described above, and which is a unit, may be compared to matter in that it likewise has a form, which is reason. If the form (reason) does not communicate its impression to the soul, then the disposition existing in the soul to receive that form is of no avail and exists to no purpose...

The “form” that may enter the human soul is intelligence ( 
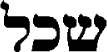
). Intelligence is a philosophical term that was often used in the middle ages. It comes to express wisdom, eternal reason coming from heaven, and appearing in the intelligent part of the soul as intelligent knowledge.

## MENTAL HEALTH LIES IN THE IMPROVEMENT OF MORALS

According to the Rambam, mental health lies in the improvement of morals. This will lead to a proper balance in the entire fabric of a human being, both mentally and physically.

The Rambam claims that the more serious mental diseases occur after important deviations from established, accepted norms in human society. The people who act contrary to the norms of human conduct, the Rambam calls “madmen” ( 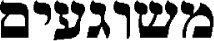). The Rambam does not treat people who are defined as such. This is his view and attitude. But today, modern medicine treats such patients by means of drugs that suppress symptoms of depression, violence, etc.

Although the leading approach in the Rambam’s era was that mental diseases are cured by correcting the patient’s morals, his books on the subject of health and medicine – in particular in his book *Chapters of Moses in Medicine* – provide information about the ingredients of drugs against depression.

About the improvement of morals, the Rambam says in the first chapter of his book *The Eight Chapters*:

Thou knowest that the improvement of the moral qualities is brought about by the healing of the soul and its activities. Therefore, just as the physician, in his endeavors to cure the human body, must have a perfect knowledge of it in its entirety and its individual parts, just as he must know what causes sickness that it may be avoided and must also be acquainted with the means by which a patient may be cured, so, likewise, he who tries to cure the soul, wishing to improve the moral qualities, must have a knowledge of the soul in its totality and its parts, must know how to prevent it from becoming diseased, and how to maintain its health.

## ABOUT DISEASES OF THE SOUL

In the Rambam’s view the body is a unit linked to the soul, yet in his philosophy he speaks about what he calls “diseases of the soul” ( 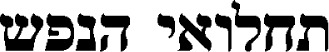). This is apparent in the third chapter of his book *The Eight Chapters*, in which he quotes the view of ancient sages ( 
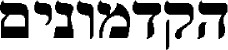
) regarding mental diseases. The Rambam mentions first of all what acts are beneficial for man to perform in order to restore his soul to health. The most striking of these acts, according to ancient sages mentioned by the Rambam, include the assimilation of noble qualities, such as engaging in charity and good deeds. It is the Rambam’s understanding that those suffering from a disease of the soul do not have such qualities and that is the source of their disease.

This is what he wrote about it in the third chapter of *The Eight Chapters*:

The ancients maintained that the soul, like the body, is subject to good health and illness. The soul’s healthful state is due to its condition and that of its faculties, by which it constantly does what is right and performs what is proper, while the illness of the soul is occasioned by its condition and that of its faculties, which results in its constantly doing wrong and performing actions that are improper. The science of medicine, however, investigates the health of the body.

The treatment for the mentally ill proposed by the Rambam is based on the same principles used in the treatment of physical diseases.

It is interesting to see how he formulates his view about this subject in the third chapter of *The Eight Chapters*:

Now, just as those who are physically ill imagine that, on account of their vitiated tastes, the sweet is bitter and the bitter is sweet – and likewise fancy the wholesome to be unwholesome – and just as their desire grows stronger and their enjoyment increases for such things as dust, coal, very acidic and sour foods, and the like – which the healthy loathe and refuse, as they are not only not beneficial even to the healthy but possibly harmful – so those whose souls are ill, that is the wicked and the morally perverted, imagine that the bad is good and the good is bad. The wicked man, moreover, continually longs for excesses, which are really pernicious but which, on account of the illness of his soul, he considers to be good.

As expected, the Rambam directs the mentally ill to turn to what he calls “the sages” and ask their advice for their health problems.

This is how the Rambam expounds his ideas in the third chapter of *The Eight Chapters*:

Likewise, just as when people, unacquainted with the science of medicine, realize that they are sick and consult a physician, who tells them what they must do, forbidding them to partake of that which they imagine beneficial, and prescribing for them things which are unpleasant and bitter in order that their bodies may become healthy and that they may again choose the good and spurn the bad, so those whose souls become ill should consult the sages, the moral physicians, who will advise them against indulging in those evils which they (the morally ill) think are good, so that they may be healed by that art of which I shall speak in the next chapter and through which the moral qualities are restored to their normal condition.

## GOOD DEEDS AND KEEPING THE GOLDEN MEAN – FORMULAS FOR MAINTAINING A HEALTHY SOUL

The Rambam’s view on how to maintain one’s mental health is a combination of both philosophical ideas and educational values. In the fourth chapter of *The Eight Chapters*, the Rambam elaborates his ideas on this interesting point.

The following are some important and interesting principles:

- Good deeds are acts performed on a basis of equilibrium, that is on the midway between two extremes. Any extreme is bad. This interesting point is expressed in the fourth chapter of his book *The Eight Chapters*: “Good deeds are such as are equilibrium, maintaining the mean between two equally bad extremes, the too much and the too little.”
- The virtues recommended to man are the following and they are all good to live by as long as the golden mean is observed: asceticism, generosity, non-covetousness, respectful behavior, courage, modesty, kindness, and patience. These, and more, are “the noble virtues of the soul of a healthy man” – in the Rambam’s words.
- Any extremism in one or more of these virtues may affect the health of one’s soul and cause a disease of the soul that may express itself in physical illness.

The Rambam is aware of the realities of life. Although in his view man is born healthy, which is “the godly way” ( 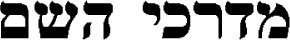), people are prone to impact in various ways by elements in their environment. Among them the Rambam mentions *inter alia* food and conduct.

Many times such impact is harmful and may corrupt one’s way of life and mental virtues. In that case he must return to the straight path – the golden mean – which is the optimal way of life for maintaining a healthy soul and body.

The Rambam is aware of the fact that no man resembles another – whether in physique, character, or the way he lives his daily life. Every person has different inner qualities. This leads to a situation in which man as an individual – mainly in a social setting – must recognize these facts and know how to act in light of the qualities of his fellow-men.

The Rambam recommends to take “the way of the righteous”, which is the straight path, and flee from extremes, since the straight path means observing the golden mean.

In this important area of mental health in connection with the body, the Rambam begins the first chapter on the Laws of Behavior (Hilchot De’ot 1, 1–2) of the volume called The Book of Knowledge of *Mishneh Torah*.

## THE BODY IN THE RAMBAM’S VIEW

The meaning and essence of the body is clear and understood. The Rambam’s vision fits in with the universal understanding of all the elements of the body’s structure and the physiological and other processes taking place in it.

Already in ancient times many details were known about the structure of the body organs and part of the physiological processes and other processes occurring in it. Many of these processes have been described – although not as thoroughly as in our modern era – by generations of Jewish sages, and also by the Rambam.

The Rambam, in his books on health and medicine, teaches man how to take care of himself and maintain health in the spirit of the concept coined by the Rambam, “the regimen of health”. The regimen of health relates to maintaining both body and soul, but a large part of the Rambam’s teachings deal with maintaining the body. Among the things man must do in order to maintain a healthy body, the Rambam mentions taking care of a proper, sensible diet and proper eating habits; physical activity – gymnastics, as the Rambam calls it; and observing other proper and sensible living habits such as sleeping hours and manner of sleeping, sexual relations, etc.

In the Rambam’s view, non-observance of proper living habits, which are based on the laws of nature, may result in diseases.

The interaction between body and soul is a wonderful combination of the body, the component which is measurable by scientific means, and the soul, the component which cannot be measured by such means. This combination is one of the corner-stones of the Rambam’s holistic view of the relation between body and soul.

The following are some interesting and important points in the connection of body and soul and their medical treatment:

According to the Rambam, the basic difference between medicine and religion is that medicine treats the body and religion treats the soul. This means: observing religious values, principles, and moral standards is the best medicine for a healthy soul. This is because the observance of moral values and integrity according to religious principles will cause a state of equilibrium in the mental faculties of man and enable him to find a positive spiritual, moral, valuable purpose for his life. Guarding these values enhances the quality and morals of their observer and enables him to live a respectful life with a rich, healthy soul, which reflects on his ability to maintain a healthy body. Therefore, the integration between medicine for the soul and medicine for the body will result in overall health. A disruption of one of those components, whether mental or physical, will result in disease.

## BODILY HEALTH IS NO LESS IMPORTANT THAN MENTAL HEALTH

The Rambam’s comprehensive, holistic approach is expressed on several levels – the philosophical, educational, and practical level.

We see that the Rambam attaches very much importance on maintaining the body no less than on maintaining the soul – which is the framework in which the body resides. Both, body and soul, constitute the essence of man.

We also see that in all the books the Rambam wrote on health and medicine, and also in *Mishneh Torah* (The Book of Knowledge, Laws of Behavior), the focus is on bodily health.

The following is the basis of the Rambam’s philosophy on the need and meaning of maintaining the body. This is how he formulates it in *Mishneh Torah* (The Book of Knowledge, Laws of Behavior 4:1):

The body being healthy is of the ways of the Lord, for it is impossible to understand or know the knowledge of the Creator while unwell. Therefore, one should keep away from things which destroy the body, and accustom oneself to healthy and curing matters, which are as follows: One should never eat unless one is hungry, nor drink unless one is thirsty, and nor should one hold oneself back for even a single moment from relieving oneself, for whenever one feels the need to pass water or to defecate, one should do so immediately.

From here, the Rambam elaborates in all his writings about foods that are recommended, the good foods”, in his terminology, and foods that are not recommended, the “bad foods”. He also explains at what hours the various foods should be eaten.

This is not the place to elaborate on the Rambam’s writings on this important subject. It is, however, clear that food is the most important issue in maintaining the body, as is apparent in *Mishneh Torah* (The Book of Knowledge, Laws of Behavior), *Regimen of Health*, and *Chapters of Moses*.

## SELECTED IMPORTANT POINTS IN THE RAMBAM’S HOLISTIC VIEWS

The following are some interesting points worth mentioning in this context:

- Maintaining health is no less important than curing a sick person. In his book *Treatise on Asthma* ( 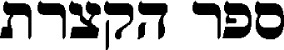), The Rambam states resolutely: “Taking care of health is required anywhere and any time, not only in times of disease but also, and in particular, in times of health.” It is the Rambam’s view that man should “persist in a regimen of health”. In our days we would call it preventive medicine!
- The approach to a person should be personal/individual, with an in-depth diagnosis of the physical, psychological, and spiritual state of the sick (or healthy) person – here and now. Relying on such personal in-depth examination, the physician will determine the treatment for his patient. In this spirit, the Rambam said: “No sick man resembles another sick man in his disease.” (*Commentary on the Aphorisms of Hippocrates*)
- The Rambam is a healer of both body and soul. The Rambam’s view of the soul and the body as one unit was also known to his fellow-physicians and sages. See for instance the comparison made by Al Sa’id Ibn Sina Maluch (an Arab physician and poet of the Rambam’s generation) between the Rambam and Galen, the Greek physician who lived a thousand years before the Rambam and was adored as a god, on their ability to cure people. This is what Ibn Sina says: “Galen was able to treat the human body, Moshe Ben-Maimon knows to bring healing to both body and soul.”

Indeed, this is proof of the Rambam’s holistic view as he considered body and soul as one unit.

## TREATING THE BODY AND TREATING THE SOUL – IMPORTANT AND INTERESTING ASPECTS IN THE RAMBAM’S VIEW

Because of the very essence of body and soul and the difference between them, treating the body and treating the soul are also different from one another.

One approach of special interest which the Rambam mentions for treating the body and treating the soul appears in the second chapter of his book *Regimen of Health*, section 2, 20. The Rambam claims that medicine of the body will strengthen what he calls the “natural force” (which is physical strength). This can only be achieved by the quality and the quantity of the food we eat.

Contrary to the body, the “vital force” (which is mental strength) can be improved by sweet fragrances like those of myrrh, amber, basil, roses, myrtle, and more.

And what is more, for strengthening the vital (mental) force, the Rambam suggests to play music near a sick person, telling him nice stories in order to “enlarge his soul and his heart”, to divert his mind and make him “laugh about them”.

## THE RAMBAM’S CRITICISM OF GALEN’S APPROACH TO MEDICINE

A striking feature in all of the Rambam’s writings is his criticism (in his writings he uses the term “conflict” ( 
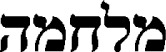
) of Galen.

This criticism was mostly focused on the fact that the healing/medical approach of Galen was centered on the body only. This criticism is expressed mainly in his books *Chapters of Moses in Medicine* and *Commentary on the Aphorisms of Hippocrates*.

## ASTHMA – AN EXAMPLE OF THE RAMBAM’S HOLISTIC APPROACH

Of all the diseases of mankind, the Rambam devotes one book especially to asthma. In this book, the Rambam advises us of his way to treat this disease. In this context he elaborates on the foods that should be consumed by asthma patients and foods that prevent asthma. Furthermore, the Rambam relates to the mental aspects linked to asthma in the eighth chapter of *Treatise on Asthma*.

In the above advice of the Rambam, one can see his holistic-comprehensive view by the fact that for treating asthma one needs to use a combination of foods, drugs, and contemplation ( 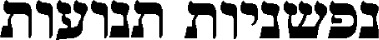) and also to “follow the path of the righteous and the prophets” ( 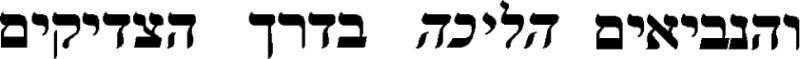). This means that one should live according to the laws of the Jewish faith, which advocates good morals and manners. All these measures together will cure asthma.

Indeed, an approach in which body and soul are one.

## EPILOGUE

### THE BODY IS THE HOME OF THE SOUL, AND THE SOUL GUIDES THE BODY

In the Rambam’s multidisciplinary ideas, he unfolds a philosophical, educational, and practical analysis of the soul and its basis, levels, and depth.

In his opinion, the body is guided by the soul. In this view, the essence of man accepts the body as a container in which the soul resides.

Except for the treatment of diseases of body and soul with drugs, foods, physical exercise, etc., the Rambam believes that maintaining health – of body and soul – lies first of all, and probably exclusively, in observing the commandments and improving one’s ways, morals, and conduct up to their highest levels, toward all of the world’s creatures. Furthermore, the Rambam is of the opinion that one needs to persist in learning the Torah. One should worship God with awe and love (in numerology His holy name is “twice love”) and observe good values and virtues. All of these build the frameworks that maintain mental health and strengthen man’s abilities to develop skills for maintaining bodily health. This is so because body and soul are one – which is the basis of the Rambam’s philosophy of health and medicine.
